# *N*-(4-Chloro­phen­yl)-2-(8-quinol­yloxy)acetamide monohydrate

**DOI:** 10.1107/S1600536810026206

**Published:** 2010-07-10

**Authors:** Yuan Wang, Yan-Wei Li, Xiao-Xia Li

**Affiliations:** a Department of Physics and Chemistry, Henan Polytechnic University, Jiaozuo 454000, People’s Republic of China; b Institute of Functional Materials, Jiangxi University of Finance & Economics, Nanchang 330013, People’s Republic of China

## Abstract

In the title compound, C_17_H_13_ClN_2_O_2_·H_2_O, the dihedral angle between the quinoline ring system and the benzene ring is 13.0 (1)°. An intra­molecular N—H⋯O hydrogen bond may influence the mol­ecular conformation. In the crystal structure, acetamide mol­ecules are linked to water mol­ecules *via* inter­molecular O—H⋯ N and N—H⋯O hydrogen bonds and in turn linked into chains along [010] *via* O—H⋯O hydrogen bonds.

## Related literature

For the synthesis of the title compound and its lanthanide complexes, see: Wu *et al.* (2008 ▶). For related structures, see: Zhang *et al.* (2006 ▶); Wu *et al.* (2010 ▶).
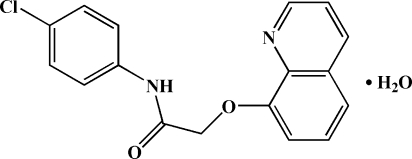


## Experimental

### 

#### Crystal data

C_17_H_13_ClN_2_O_2_·H_2_O
*M* *_r_* = 330.76
Orthorhombic, 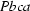

*a* = 19.4984 (19) Å
*b* = 5.2601 (6) Å
*c* = 29.851 (3) Å
*V* = 3061.7 (5) Å^3^
*Z* = 8
Mo *K*α radiation
μ = 0.27 mm^−1^
*T* = 296 K
0.32 × 0.23 × 0.20 mm

#### Data collection

Bruker SMART CCD diffractometer
Absorption correction: multi-scan (*SADABS*; Sheldrick, 1996 ▶) *T*_min_ = 0.929, *T*_max_ = 0.948
15222 measured reflections
3622 independent reflections
1800 reflections with *I* > 2σ(*I*)
*R*_int_ = 0.050

#### Refinement

*R*[*F* ^2^ > 2σ(*F* ^2^)] = 0.047
*wR*(*F* ^2^) = 0.128
*S* = 1.00
3622 reflections
217 parameters
4 restraints
H atoms treated by a mixture of independent and constrained refinement
Δρ_max_ = 0.16 e Å^−3^
Δρ_min_ = −0.21 e Å^−3^

### 

Data collection: *SMART* (Bruker, 1997 ▶); cell refinement: *SAINT* (Bruker, 1997 ▶); data reduction: *SAINT*; program(s) used to solve structure: *SHELXS97* (Sheldrick, 2008 ▶); program(s) used to refine structure: *SHELXL97* (Sheldrick, 2008 ▶); molecular graphics: *SHELXTL* (Sheldrick, 2008 ▶); software used to prepare material for publication: *SHELXTL*.

## Supplementary Material

Crystal structure: contains datablocks I, global. DOI: 10.1107/S1600536810026206/lh5076sup1.cif

Structure factors: contains datablocks I. DOI: 10.1107/S1600536810026206/lh5076Isup2.hkl

Additional supplementary materials:  crystallographic information; 3D view; checkCIF report

## Figures and Tables

**Table 1:** Hydrogen-bond geometry (Å, °)

*D*—H⋯*A*	*D*—H	H⋯*A*	*D*⋯*A*	*D*—H⋯*A*
O1*W*—H1*WA*⋯O1*W*^i^	0.85 (1)	2.06 (1)	2.9014 (16)	168 (2)
O1*W*—H1*WB*⋯N2	0.85 (1)	1.99 (1)	2.830 (2)	170 (2)
N1—H1*A*⋯O2	0.83 (1)	2.27 (2)	2.702 (2)	113 (2)
N1—H1*A*⋯O1*W*	0.83 (1)	2.40 (2)	3.088 (2)	140 (2)

Symmetry code: (i) 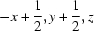.

**Fig. 1.**
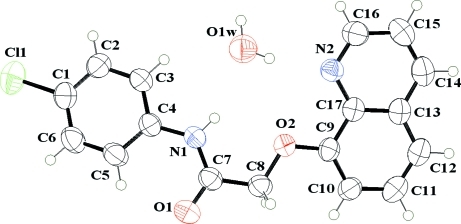
The molecular structure shown with 50% probability displacement ellipsoids.

**Fig. 2.**
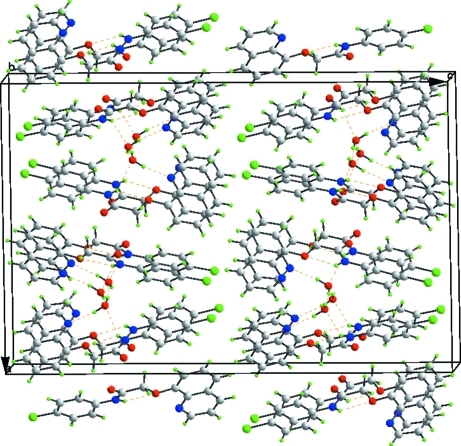
Part of the crystal structure viewed approximately along the b axis with hydrogen bonds shown as dashed lines.

## supplementary crystallographic information

### Comment

Amide type ligands have been extensively investigated due to their excellent coordination abilities (Wu *et al.*, 2008;2010). As part of our ongoing studies of amide type ligands, the title compound was synthesized and characterized by X-ray diffraction.

In the title compound, all the bond lengths are comparable with those observed in a similar compound (Zhang *et al.*, 2006). The dihedral angle between quinoline ring (N2/C9–C17, r.m.s. deviation 0.0129 Å) and benzene ring (C1–C6, r.m.s. deviation 0.0008 Å) is 13.0 (1)°. An intramolecular N-H···O hydrogen bond may influence the molecular conformation. In the crystal structure, *N*-(4-chlorophenyl)-2- (quinolin-8-yloxy)acetamide molecules are linked to water molecules *via* intermolecular O—H··· N and N—H···O hydrogen bonds and in turn linked into one-dimensional chains along [010] via O-H···O hydrogen bonds.  Additional stabilization is provided by weak π···π stacking interactions involving the benzene ring and pyridine rings of symmetry related quinoline groups with a centroid to centroid distance of 3.8607 (14) Å.

### Experimental

The title compound was prepared according to the literature, Wu *et al.* (2008). Colorless block crystals were obtained by slow evaporation of a *N*,*N*-dimethylformamide solution of the title compound.

### Refinement

The N—H and water H-atoms were located in a difference Fourier map and refined with an N—H distance restraint of 0.83 (1)Å and an O—H distance restraint of 0.85 (1)Å. H atoms attached to C atoms were placed in calculated positions and treated using a riding-model approximation (C—H = 0.93; *U*_iso_(H)=1.2U_eq_(C)).

### Crystal data

**Table:**

C_17_H_13_ClN_2_O_2_·H_2_O	*F*(000) = 1376
*M**_r_* = 330.76	*D*_x_ = 1.435 Mg m^−^^3^
Orthorhombic, *P**b**c**a*	Mo *K*α radiation, λ = 0.71073 Å
Hall symbol: -P 2ac 2ab	Cell parameters from 1957 reflections
*a* = 19.4984 (19) Å	θ = 2.5–19.9°
*b* = 5.2601 (6) Å	µ = 0.27 mm^−^^1^
*c* = 29.851 (3) Å	*T* = 296 K
*V* = 3061.7 (5) Å^3^	Block, colorless
*Z* = 8	0.32 × 0.23 × 0.20 mm

### Data collection

**Table:**

Bruker SMART CCD diffractometer	3622 independent reflections
Radiation source: sealed tube	1800 reflections with *I* > 2σ(*I*)
graphite	*R*_int_ = 0.050
φ and ω scans	θ_max_ = 28.1°, θ_min_ = 1.4°
Absorption correction: multi-scan (*SADABS*; Sheldrick, 1996)	*h* = −18→25
*T*_min_ = 0.929, *T*_max_ = 0.948	*k* = −6→4
15222 measured reflections	*l* = −39→39

### Refinement

**Table:**

Refinement on *F*^2^	Primary atom site location: structure-invariant direct methods
Least-squares matrix: full	Secondary atom site location: difference Fourier map
*R*[*F*^2^ > 2σ(*F*^2^)] = 0.047	Hydrogen site location: inferred from neighbouring sites
*wR*(*F*^2^) = 0.128	H atoms treated by a mixture of independent and constrained refinement
*S* = 1.00	*w* = 1/[σ^2^(*F*_o_^2^) + (0.0548*P*)^2^ + 0.0231*P*] where *P* = (*F*_o_^2^ + 2*F*_c_^2^)/3
3622 reflections	(Δ/σ)_max_ < 0.001
217 parameters	Δρ_max_ = 0.16 e Å^−^^3^
4 restraints	Δρ_min_ = −0.21 e Å^−^^3^

### Special details

**Table:**

Geometry. All e.s.d.'s (except the e.s.d. in the dihedral angle between two l.s. planes) are estimated using the full covariance matrix. The cell e.s.d.'s are taken into account individually in the estimation of e.s.d.'s in distances, angles and torsion angles; correlations between e.s.d.'s in cell parameters are only used when they are defined by crystal symmetry. An approximate (isotropic) treatment of cell e.s.d.'s is used for estimating e.s.d.'s involving l.s. planes.
Refinement. Refinement of *F*^2^ against ALL reflections. The weighted *R*-factor *wR* and goodness of fit *S* are based on *F*^2^, conventional *R*-factors *R* are based on *F*, with *F* set to zero for negative *F*^2^. The threshold expression of *F*^2^ > σ(*F*^2^) is used only for calculating *R*-factors(gt) *etc*. and is not relevant to the choice of reflections for refinement. *R*-factors based on *F*^2^ are statistically about twice as large as those based on *F*, and *R*- factors based on ALL data will be even larger.

### Fractional atomic coordinates and isotropic or equivalent isotropic displacement parameters (Å^2^)

**Table:**

	*x*	*y*	*z*	*U*_iso_*/*U*_eq_	
Cl1	0.32260 (4)	0.36909 (15)	0.044180 (19)	0.0858 (3)	
O2	0.40858 (7)	0.1726 (3)	0.32297 (4)	0.0532 (4)	
N2	0.34502 (9)	0.5390 (3)	0.36817 (6)	0.0505 (5)	
N1	0.38877 (10)	0.1567 (3)	0.23341 (6)	0.0524 (5)	
C10	0.47572 (12)	0.0589 (4)	0.38814 (7)	0.0568 (6)	
H10	0.4991	−0.0675	0.3726	0.068*	
C17	0.39211 (11)	0.3959 (4)	0.39023 (6)	0.0454 (5)	
C13	0.40699 (12)	0.4337 (4)	0.43606 (7)	0.0522 (6)	
C7	0.42016 (11)	−0.0454 (4)	0.25142 (7)	0.0506 (6)	
C9	0.42715 (11)	0.2012 (4)	0.36669 (6)	0.0470 (5)	
C11	0.49027 (12)	0.1029 (5)	0.43328 (8)	0.0647 (7)	
H11	0.5238	0.0060	0.4474	0.078*	
C12	0.45676 (13)	0.2827 (4)	0.45688 (7)	0.0618 (7)	
H12	0.4667	0.3065	0.4871	0.074*	
C14	0.37041 (14)	0.6238 (5)	0.45851 (7)	0.0650 (7)	
H14	0.3782	0.6522	0.4888	0.078*	
O1	0.43645 (9)	−0.2349 (3)	0.23095 (5)	0.0771 (5)	
C1	0.34138 (12)	0.3011 (5)	0.09954 (7)	0.0564 (6)	
C8	0.43823 (12)	−0.0380 (4)	0.30005 (7)	0.0529 (6)	
H8A	0.4228	−0.1942	0.3141	0.063*	
H8B	0.4877	−0.0298	0.3030	0.063*	
C4	0.37288 (11)	0.1944 (4)	0.18780 (7)	0.0480 (5)	
C3	0.33244 (12)	0.3998 (4)	0.17682 (7)	0.0569 (6)	
H3	0.3155	0.5039	0.1995	0.068*	
C2	0.31662 (12)	0.4537 (5)	0.13280 (7)	0.0612 (6)	
H2	0.2893	0.5930	0.1258	0.073*	
C16	0.31355 (12)	0.7171 (5)	0.39060 (7)	0.0619 (6)	
H16	0.2822	0.8181	0.3753	0.074*	
C6	0.38144 (13)	0.0964 (5)	0.10979 (7)	0.0649 (7)	
H6	0.3982	−0.0070	0.0870	0.078*	
C5	0.39718 (13)	0.0424 (4)	0.15377 (7)	0.0629 (7)	
H5	0.4244	−0.0977	0.1605	0.075*	
C15	0.32395 (14)	0.7662 (5)	0.43632 (8)	0.0681 (7)	
H15	0.2995	0.8934	0.4509	0.082*	
O1W	0.28141 (9)	0.4811 (3)	0.28357 (5)	0.0696 (5)	
H1A	0.3792 (14)	0.272 (4)	0.2514 (7)	0.104*	
H1WA	0.2599 (13)	0.621 (3)	0.2800 (7)	0.104*	
H1WB	0.3043 (12)	0.488 (5)	0.3078 (6)	0.104*	

### Atomic displacement parameters (Å^2^)

**Table:**

	*U*^11^	*U*^22^	*U*^33^	*U*^12^	*U*^13^	*U*^23^
Cl1	0.0970 (6)	0.1067 (6)	0.0538 (4)	−0.0048 (5)	−0.0072 (3)	0.0087 (4)
O2	0.0583 (10)	0.0571 (10)	0.0443 (8)	0.0109 (8)	−0.0004 (7)	−0.0023 (7)
N2	0.0479 (11)	0.0527 (11)	0.0509 (10)	0.0024 (10)	0.0006 (9)	−0.0023 (9)
N1	0.0637 (13)	0.0479 (12)	0.0455 (11)	0.0084 (10)	0.0035 (9)	−0.0050 (9)
C10	0.0559 (15)	0.0596 (15)	0.0548 (13)	0.0078 (13)	0.0008 (11)	0.0075 (11)
C17	0.0428 (13)	0.0474 (13)	0.0460 (12)	−0.0093 (11)	0.0013 (10)	0.0031 (10)
C13	0.0564 (16)	0.0522 (14)	0.0479 (13)	−0.0131 (12)	0.0007 (11)	0.0020 (11)
C7	0.0553 (15)	0.0433 (13)	0.0533 (13)	−0.0019 (12)	0.0051 (11)	−0.0029 (11)
C9	0.0467 (13)	0.0514 (14)	0.0431 (11)	−0.0045 (11)	0.0017 (10)	0.0048 (10)
C11	0.0607 (17)	0.0717 (17)	0.0618 (15)	0.0002 (14)	−0.0119 (13)	0.0128 (13)
C12	0.0712 (18)	0.0652 (17)	0.0490 (13)	−0.0091 (14)	−0.0092 (13)	0.0060 (12)
C14	0.080 (2)	0.0656 (17)	0.0492 (14)	−0.0122 (15)	0.0029 (13)	−0.0056 (12)
O1	0.1077 (15)	0.0544 (11)	0.0691 (10)	0.0222 (10)	−0.0111 (10)	−0.0142 (9)
C1	0.0581 (16)	0.0621 (16)	0.0490 (13)	−0.0105 (13)	0.0011 (11)	0.0007 (12)
C8	0.0611 (16)	0.0450 (13)	0.0525 (13)	0.0043 (12)	0.0072 (11)	0.0048 (11)
C4	0.0503 (15)	0.0452 (12)	0.0485 (12)	−0.0055 (11)	0.0031 (11)	−0.0035 (10)
C3	0.0629 (16)	0.0520 (14)	0.0558 (14)	0.0068 (13)	−0.0027 (12)	−0.0110 (11)
C2	0.0615 (15)	0.0600 (15)	0.0621 (15)	0.0043 (13)	−0.0094 (13)	−0.0010 (12)
C16	0.0584 (16)	0.0599 (15)	0.0673 (15)	0.0036 (13)	−0.0009 (13)	−0.0050 (13)
C6	0.0829 (19)	0.0631 (16)	0.0486 (14)	0.0028 (15)	0.0136 (13)	−0.0053 (12)
C5	0.0821 (18)	0.0527 (14)	0.0539 (14)	0.0125 (13)	0.0141 (13)	−0.0024 (11)
C15	0.0737 (19)	0.0673 (17)	0.0634 (16)	−0.0007 (15)	0.0090 (14)	−0.0175 (14)
O1W	0.0808 (14)	0.0720 (13)	0.0560 (10)	0.0091 (10)	−0.0059 (9)	−0.0056 (9)

### Geometric parameters (Å, °)

**Table:**

Cl1—C1	1.730 (2)	C14—C15	1.349 (3)
O2—C9	1.363 (2)	C14—H14	0.9300
O2—C8	1.425 (2)	C1—C6	1.365 (3)
N2—C16	1.305 (3)	C1—C2	1.365 (3)
N2—C17	1.358 (2)	C8—H8A	0.9700
N1—C7	1.339 (3)	C8—H8B	0.9700
N1—C4	1.411 (3)	C4—C5	1.377 (3)
N1—H1A	0.832 (10)	C4—C3	1.377 (3)
C10—C9	1.366 (3)	C3—C2	1.379 (3)
C10—C11	1.396 (3)	C3—H3	0.9300
C10—H10	0.9300	C2—H2	0.9300
C17—C13	1.412 (3)	C16—C15	1.404 (3)
C17—C9	1.417 (3)	C16—H16	0.9300
C13—C12	1.400 (3)	C6—C5	1.378 (3)
C13—C14	1.399 (3)	C6—H6	0.9300
C7—O1	1.212 (2)	C5—H5	0.9300
C7—C8	1.494 (3)	C15—H15	0.9300
C11—C12	1.348 (3)	O1W—H1WA	0.854 (9)
C11—H11	0.9300	O1W—H1WB	0.851 (9)
C12—H12	0.9300		
			
C9—O2—C8	115.98 (16)	C6—C1—Cl1	119.91 (18)
C16—N2—C17	117.87 (19)	C2—C1—Cl1	119.9 (2)
C7—N1—C4	126.84 (18)	O2—C8—C7	113.02 (17)
C7—N1—H1A	115.0 (19)	O2—C8—H8A	109.0
C4—N1—H1A	118.2 (19)	C7—C8—H8A	109.0
C9—C10—C11	120.1 (2)	O2—C8—H8B	109.0
C9—C10—H10	119.9	C7—C8—H8B	109.0
C11—C10—H10	119.9	H8A—C8—H8B	107.8
N2—C17—C13	122.04 (19)	C5—C4—C3	118.5 (2)
N2—C17—C9	119.08 (18)	C5—C4—N1	123.7 (2)
C13—C17—C9	118.88 (19)	C3—C4—N1	117.76 (19)
C12—C13—C14	123.1 (2)	C4—C3—C2	121.1 (2)
C12—C13—C17	119.5 (2)	C4—C3—H3	119.5
C14—C13—C17	117.4 (2)	C2—C3—H3	119.5
O1—C7—N1	124.8 (2)	C1—C2—C3	119.6 (2)
O1—C7—C8	116.7 (2)	C1—C2—H2	120.2
N1—C7—C8	118.50 (19)	C3—C2—H2	120.2
O2—C9—C10	124.9 (2)	N2—C16—C15	124.3 (2)
O2—C9—C17	115.25 (18)	N2—C16—H16	117.9
C10—C9—C17	119.84 (19)	C15—C16—H16	117.9
C12—C11—C10	121.5 (2)	C1—C6—C5	120.3 (2)
C12—C11—H11	119.3	C1—C6—H6	119.9
C10—C11—H11	119.3	C5—C6—H6	119.9
C11—C12—C13	120.1 (2)	C4—C5—C6	120.4 (2)
C11—C12—H12	119.9	C4—C5—H5	119.8
C13—C12—H12	119.9	C6—C5—H5	119.8
C15—C14—C13	120.3 (2)	C14—C15—C16	118.2 (2)
C15—C14—H14	119.9	C14—C15—H15	120.9
C13—C14—H14	119.9	C16—C15—H15	120.9
C6—C1—C2	120.2 (2)	H1WA—O1W—H1WB	109.2 (15)
			
C16—N2—C17—C13	0.5 (3)	C12—C13—C14—C15	178.8 (2)
C16—N2—C17—C9	−179.92 (19)	C17—C13—C14—C15	−1.2 (3)
N2—C17—C13—C12	−179.1 (2)	C9—O2—C8—C7	−175.30 (17)
C9—C17—C13—C12	1.3 (3)	O1—C7—C8—O2	−171.25 (19)
N2—C17—C13—C14	0.9 (3)	N1—C7—C8—O2	10.0 (3)
C9—C17—C13—C14	−178.67 (19)	C7—N1—C4—C5	−10.4 (4)
C4—N1—C7—O1	−4.3 (4)	C7—N1—C4—C3	171.6 (2)
C4—N1—C7—C8	174.31 (19)	C5—C4—C3—C2	−0.2 (3)
C8—O2—C9—C10	6.1 (3)	N1—C4—C3—C2	177.9 (2)
C8—O2—C9—C17	−174.00 (17)	C6—C1—C2—C3	0.1 (3)
C11—C10—C9—O2	−179.4 (2)	Cl1—C1—C2—C3	−179.29 (18)
C11—C10—C9—C17	0.8 (3)	C4—C3—C2—C1	0.0 (3)
N2—C17—C9—O2	−1.3 (3)	C17—N2—C16—C15	−1.7 (3)
C13—C17—C9—O2	178.33 (18)	C2—C1—C6—C5	0.0 (4)
N2—C17—C9—C10	178.64 (19)	Cl1—C1—C6—C5	179.38 (19)
C13—C17—C9—C10	−1.8 (3)	C3—C4—C5—C6	0.3 (3)
C9—C10—C11—C12	0.7 (4)	N1—C4—C5—C6	−177.6 (2)
C10—C11—C12—C13	−1.2 (4)	C1—C6—C5—C4	−0.2 (4)
C14—C13—C12—C11	−179.9 (2)	C13—C14—C15—C16	0.2 (4)
C17—C13—C12—C11	0.1 (3)	N2—C16—C15—C14	1.3 (4)

### Hydrogen-bond geometry (Å, °)

**Table:**

*D*—H···*A*	*D*—H	H···*A*	*D*···*A*	*D*—H···*A*
O1W—H1WA···O1W^i^	0.85 (1)	2.06 (1)	2.9014 (16)	168 (2)
N1—H1A···O2	0.83 (1)	2.27 (2)	2.702 (2)	113 (2)
N1—H1A···O1W	0.83 (1)	2.40 (2)	3.088 (2)	140 (2)
O1W—H1WB···N2	0.85 (1)	1.99 (1)	2.830 (2)	170 (2)

Symmetry codes: (i) −*x*+1/2, *y*+1/2, *z*.
